# Basins of attraction of chimera states on networks

**DOI:** 10.3389/fphys.2022.959431

**Published:** 2022-09-08

**Authors:** Qiang Li, Kelly C. Larosz, Dingding Han, Peng Ji, Jürgen Kurths

**Affiliations:** ^1^ Institute of Science and Technology for Brain-Inspired Intelligence, Fudan University, Shanghai, China; ^2^ Key Laboratory of Computational Neuroscience and Brain-Inspired Intelligence (Fudan University), Ministry of Education, Shanghai, China; ^3^ MOE Frontiers Center for Brain Science, Fudan University, Shanghai, China; ^4^ University Center UNIFATEB, Telêmaco Borba, Brazil; ^5^ Graduate Program in Chemical Engineering Federal Technological University of Paraná, Ponta Grossa, Brazil; ^6^ Physics Institute, University of São Paulo, Oscillation Control Group, São Paulo, Brazil; ^7^ School of Information Science and Technology, Fudan University, Shanghai, China; ^8^ Research Institute of Intelligent Complex Systems, Fudan University, Shanghai, China; ^9^ Potsdam Institute for Climate Impact Research, Potsdam, Germany; ^10^ Humboldt University, Berlin, Germany

**Keywords:** complex networks, synchronization patterns, basin stability, chimera states, brain network

## Abstract

Networks of identical coupled oscillators display a remarkable spatiotemporal pattern, the chimera state, where coherent oscillations coexist with incoherent ones. In this paper we show quantitatively in terms of basin stability that stable and breathing chimera states in the original two coupled networks typically have very small basins of attraction. In fact, the original system is dominated by periodic and quasi-periodic chimera states, in strong contrast to the model after reduction, which can not be uncovered by the Ott-Antonsen ansatz. Moreover, we demonstrate that the curve of the basin stability behaves bimodally after the system being subjected to even large perturbations. Finally, we investigate the emergence of chimera states in brain network, through inducing perturbations by stimulating brain regions. The emerged chimera states are quantified by Kuramoto order parameter and chimera index, and results show a weak and negative correlation between these two metrics.

## 1 Introduction

The Kuramoto model is known to exhibit various complex phenomena of collective synchronization, where nonidentical oscillators spontaneously lock in a common frequency, except those with very different natural frequency (Arenas et al., 2008). Identical oscillators, however, were expected to display simple collective behaviors until the discovery of a chimera state (Kuramoto and Battogtokh, 2002; Abrams and Strogatz, 2004). The chimera state is a spatiotemporal pattern where a network of coupled oscillators is split into coexisting subpopulations of synchronized and desynchronized oscillations (Kuramoto and Battogtokh, 2002; Abrams and Strogatz, 2004). It has been observed theoretically in networks of general types of oscillators (Abrams et al., 2008; Pikovsky and Rosenblum, 2008; Panaggio and Abrams, 2015), as well as in experiments including chemical systems (Hagerstrom et al., 2012; Tinsley et al., 2012; Nkomo et al., 2013) and mechanical oscillators (Martens et al., 2013). In biology, the chimera state is observed in Wilson-Cowan oscillators (WCOs), which obey a nonlinear mean-field model to describe the dynamics of brain network (Wilson and Cowan, 1972; Bansal et al., 2019).

Mathematical studies of chimera states have focused on a special class of density function based on the Ott-Antonsen ansatz and have analytically described stable and breathing chimera states (Abrams et al., 2008). *Via* the Watanabe-Strogatz theory, governing equations are reduced to low-dimensional systems with only three transformed parameters (Pikovsky and Rosenblum, 2008), which complements the results of chimera states (Abrams et al., 2008). This ansatz is very efficient for a network of Kuramoto oscillators. Note that chimera states are stable, persistent phenomena for *N* → *∞* (Omel’chenko, 2013) and are sensitive to perturbations with typically small basins of attraction. Therefore, it is crucial to investigate the stability of chimera states against perturbations. Basin stability indicates the likelihood that the system (or a group) will retain a desirable state after being subjected to even large perturbations and is calculated proportional to the volume of the basin of attraction of the desirable state (Menck et al., 2013).

In addition to the mathematical studies, chimera states in brain networks have also received great attentions to deepen the understanding of cognitive function (input, integration and output), from various perspectives. From dynamical perspectives, networked FitzHugh-Nagumo oscillators could induce chimera states for certain range of coupling strengths (Chouzouris et al., 2018). Adaptive couplings could also yield a self-organized state and induce chimera states (Huo et al., 2019). From structural perspectives, an empirical brain network is applied to explore how brain structures impact chimera states through numerical disruptions (Bansal et al., 2019). The two-layer brain network reproduces the phenomena of unihemispheric sleep with one hemisphere synchronized and the other desynchronized (Kang et al., 2019). This further explains the first-night effect in human sleep (Tamaki et al., 2016). These results could be further utilized to analyze the mechanism of brain functions, e.g., cognition and memory, and so on (Wang and Liu, 2020; Parastesh et al., 2021).

In this paper, we investigate the stability of chimera states of the coupled networks by using the coupling scheme of Ref. (Abrams et al., 2008), where two populations are fully connected but with different intra- and inter-coupling strengths, by means of basin stability. We first analyze the stability of the low-dimensional model after the phase reduction using the Ott-Antonsen ansatz and approximate basin stability of chimera states in terms of their attracting basins. In comparison to the model after reduction, we substantially perturb the original dynamics in the coupled networks. In this way, instead of stable or breathing chimera states, we quantitatively show that the original system is dominated by periodic or quasi-periodic chimera states. We also observe that after the system being subjected to even more and large perturbations, the curve of basin stability of the chimera states behaves bimodally. To investigate how chimera states in brain are influenced by stimulation of various brain regions, we integrate WCOs on brain networks and stimulate single region with three global coupling strengths. Results show the existing of three different states, i.e., the coherent, chimera, and metastable state (Yeo et al., 2011; Dimulescu et al., 2021), and suggest that the Kuramoto order parameter is weakly and negatively correlated with the chimera index. Besides, higher degree nodes have a more centralized and compact distribution of the ranked order parameter compared to lower ones.

## 2 Model

In this paper, we focus on chimera states in theoretical and applied aspects, with Kuramoto model by dimensional reduction and the coupled Wilson-Cowan oscillators on brain networks.

The governing equations for Kuramoto model follow (Abrams et al., 2008)

$$\frac{d\theta_{i}^{\sigma }}{dt}=\omega +\overset{2}{\underset{\sigma^{\prime }=1}{\sum }}\frac{K_{\sigma \sigma^{\prime }}}{N_{\sigma^{\prime }}}\overset{N_{\sigma^{\prime }}}{\underset{j=1}{\sum }}\sin\left(\theta_{j}^{\sigma^{\prime }}-\theta_{i}^{\sigma }-\alpha \right),$$ (1)

where $\theta_{i}^{\sigma }$ is the phase of the *i*-th oscillator (*i* = 1, …, *N*_*σ*_) in the population indicated by *σ* = 1, 2. *N*_*σ*_ denotes the number of oscillators in *σ*. The oscillators are assumed to be identical with the same natural frequency *ω* and the same phase lag *α*, and they are globally coupled either with the intra-coupling strength *K*_11_ = *K*_22_ = *μ* > 0 within the same population or with the inter-coupling strength *K*_12_ = *K*_21_ = *ν* > 0 between different populations. The intra-coupling is stronger than the inter-coupling, i.e., *μ* > *ν*. We set *μ* + *ν* = 1, the coupling disparity *A* = *μ* − *ν*, and *β* = *π*/2 − *α*.

The dynamics on brain behavior are modeled by Wilson-Cowan oscillators (WCOs), describing the evolution of excitatory and inhibitory activity in a coupled brain network (Wilson and Cowan, 1972; Bansal et al., 2019). In particular, we consider a brain network with *N* brain regions, the connection strength between brain regions *i* and *j* accounted for by *A*_*ij*_. At time *t*, we use *E*_*i*_(*t*) and *I*_*i*_(*t*) to denote the fraction of excitatory and inhibitory neurons activities, respectively, in the *i*-th brain region. The temporal dynamics of *E*_*i*_(*t*) and *I*_*i*_(*t*) are governed by

$$\begin{array}{l} \begin{array}{cc} \tau \frac{dE_{i}\left(t\right)}{dt} & =-E_{i}\left(t\right)+\left(S_{E_{m}}-E_{i}\left(t\right)\right)XS_{E}\left(c_{1}E_{i}\left(t\right)-c_{2}I_{i}\left(t\right)+c_{5}\underset{j}{\sum }A_{ij}E_{j}\left(t-\tau_{d}^{ij}\right)+P_{i}\left(t\right)\right)+\eta w_{i}\left(t\right),\\ \tau \frac{dI_{i}}{dt} & =-I_{i}\left(t\right)+\left(S_{I_{m}}-I_{i}\left(t\right)\right)XS_{I}\left(c_{3}E_{i}\left(t\right)-c_{4}I_{i}\left(t\right)+c_{6}\underset{j}{\sum }A_{ij}I_{j}\left(t-\tau_{d}^{ij}\right)\right)+\eta v_{i}\left(t\right),\;\;i=1,\ldots ,N, \end{array} \end{array}$$ (2)

where

$$S_{E,I}\left(x\right)=\frac{1}{1+e^{-a_{E,I}\left(x-\theta_{E,I}\right)}}-\frac{1}{1+e^{a_{E,I}\theta_{E,I}}},$$

with the maximal values $S_{E_{m}}$ and $S_{I_{m}}$ .

In Eq. 2, the parameters *c*_5_ and *c*_6_ represent the excitatory and inhibitory global coupling strength between brain regions, respectively, with *c*_6_ = *c*_5_/4. The term *P*_*i*_(*t*) determines the external stimulation to excitatory neurons activities. The parameter $\tau_{d}^{ij}$ represents the communication delay from regions *j* to *i*. The spatial distance *d*_*ij*_ corresponds to the communication delay as $\tau_{d}^{ij}=d_{ij}/t_{d}$ , with the signal transmission velocity *t*_*d*_ = 10 m/s. White noises, *w*_*i*_(*t*) and *v*_*i*_(*t*), are generated from a normal distribution with standard deviation *η* = 0.001. Other parameters are biologically derived, *c*_1_ = 16, *c*_2_ = 12, *c*_3_ = 15, *c*_4_ = 3, *a*_*E*_ = 1.3, *a*_*I*_ = 2, *θ*_*E*_ = 4, *θ*_*I*_ = 3.7, and *τ* = 8 (Wilson and Cowan, 1972; Muldoon et al., 2016; Bansal et al., 2018; Bansal et al., 2019).

To quantitatively analyze the degree of synchronization of the population *σ*, we consider the complex order parameter

$$r_{\sigma }e^{\text{i}\psi_{\sigma }}=\frac{1}{N_{\sigma }}\overset{N_{\sigma }}{\underset{j=1}{\sum }}e^{\text{i}\theta_{j}^{\sigma }},$$ (3)

as a macroscopic quantity, where $\text{i}=\sqrt{-1}$ , *r*_*σ*_ measures the instantaneous phase coherence and *ψ*_*σ*_ indicates the average phase in *σ*. Considering the continuum limit where *N*_*σ*_ → *∞*, the state of the population *σ* at time *t* is described by the probability density function *f*_*σ*_(*θ* ^*σ*^, *t*) with $\overset{2\pi }{\underset{0}{\int }}f_{\sigma }\left(\theta^{\sigma },t\right)d\theta^{\sigma }=1$ . This yields

$$r_{\sigma }e^{\text{i}\psi_{\sigma }}=\int e^{\text{i}\theta^{\sigma }}f_{\sigma }\left(\theta^{\sigma },t\right)d\theta^{\sigma }.$$ (4)

In what follows, we focus on the emergence of chimera states from the theoretical derivations and numerical investigation on an empirical brain network. The theoretical part provides the quantitatively basin stability of the chimera state, as well as comparing the difference of attracting basins of chimera states between the original system and the reductional system. The applied part provides the investigation of the impact of stimulating single region on the brain dynamics and how the induced chimera states are influenced by the stimulation of various regions.

## 3 Low-dimensional system

To analytically investigate the dynamics, stability and bifurcations of the system (1), it is convenient to explore a reduction of the phase model to a low-dimensional description of each population (Abrams et al., 2008) in terms of the ansatz imposed by Ott and Antonsen (Ott and Antonsen, 2008). Previous studies of chimera states focused on a special class of density function based on the Ott-Antonsen ansatz and have analytically described stable and breathing chimera states (Abrams et al., 2008). In this paper, we use a special Poisson kernel density function based on the Ott-Antonsen ansatz and obtain a reduced system of the original Kuramoto system. The reduced system is derived to provide the quantitatively basin stability of the chimera state, and to compare the difference of attracting basins of chimera states between the original systems and the reduced system.

Assuming that the density function *f*_*σ*_(*θ* ^*σ*^, *t*) follows a special Poisson kernel and expanding *f*_*σ*_(*θ* ^*σ*^, *t*) in a Fourier series in *θ* ^*σ*^, we have

$$f_{\sigma }\left(\theta^{\sigma },t\right)=\frac{1}{2\pi }\left\{1+\left[\overset{\infty }{\underset{n=1}{\sum }}{\left[a_{\sigma }\left(t\right)e^{\text{i}\theta^{\sigma }}\right]}^{n}+c.c.\right]\right\},$$ (5)

where *c*.*c*. Stands for complex conjugate and |*a*_*σ*_(*t*)| ≤ 1 to avoid divergence. Substituting the Fourier expansion Eq. 5 of the density function into the order parameter Eq. 4 yields

$$r_{\sigma }e^{\text{i}\psi_{\sigma }}=a_{\sigma }^{*}\left(t\right),$$ (6)

where $a_{\sigma }^{*}\left(t\right)$ denote the complex conjugate of the Fourier coefficient *a*_*σ*_(*t*).

In the limit *N*_*σ*_ → *∞*, we can get a reduction of the governing Eq. 1. Recall that *f*_*σ*_ satisfies the continuity equation $\frac{\partial f_{\sigma }}{\partial t}+\frac{\partial f_{\sigma }v^{\sigma }}{\partial \theta^{\sigma }}=0$ , where *v* ^*σ*^ is the phase velocity and is determined by the right side of Eq. 1. Inserting the Fourier expansion (5) of *f*_*σ*_ into the continuity equation, one can reproduce the amplitude equations (for more details in Ref. (Abrams et al., 2008)).

The amplitude equations can be rewritten in terms of polar coordinates *r*_*σ*_ and *ψ*_*σ*_ according to Eq. 6 and obtain a two-dimensional system given by

$$\begin{array}{cc} {\dot{r}}_{1} & =\frac{1-r_{1}^{2}}{2}\left(\mu r_{1}\cos\left(a\right)+\nu r_{2}\cos\left(\psi +a\right)\right),\\ {\dot{r}}_{2} & =\frac{1-r_{2}^{2}}{2}\left(\mu r_{2}\cos\left(a\right)+\nu r_{1}\cos\left(\psi -a\right)\right),\\ \dot{\psi } & =\frac{1+r_{2}^{2}}{2r_{2}}\left(\mu r_{2}\sin\left(a\right)+\nu r_{1}\sin\left(-\psi +a\right)\right)-\frac{1+r_{1}^{2}}{2r_{1}}\left(\mu r_{1}\sin\left(a\right)+\nu r_{2}\sin\left(\psi +a\right)\right), \end{array}$$ (7)

where *ψ* = *ψ*_1_ − *ψ*_2_.

We have investigated the behavior of this low-dimensional system Eq. 7. The linear stability analysis of the system has been well performed (Abrams et al., 2008), but their attracting basins was not studied. In particular, the basins of attraction of chimera states have not attracted great attention (Panaggio and Abrams, 2015). Basins of attraction of Chimera states of a simple system of two populations have been investigated using perturbative analysis Martens et al. (2016). Following the similar notation of symbols (e.g., 1S2D) of the reference Martens et al. (2016), we analyze the stability diagram *A* and *β* of chimera states in low dimensional systems.

**FIGURE 1:**
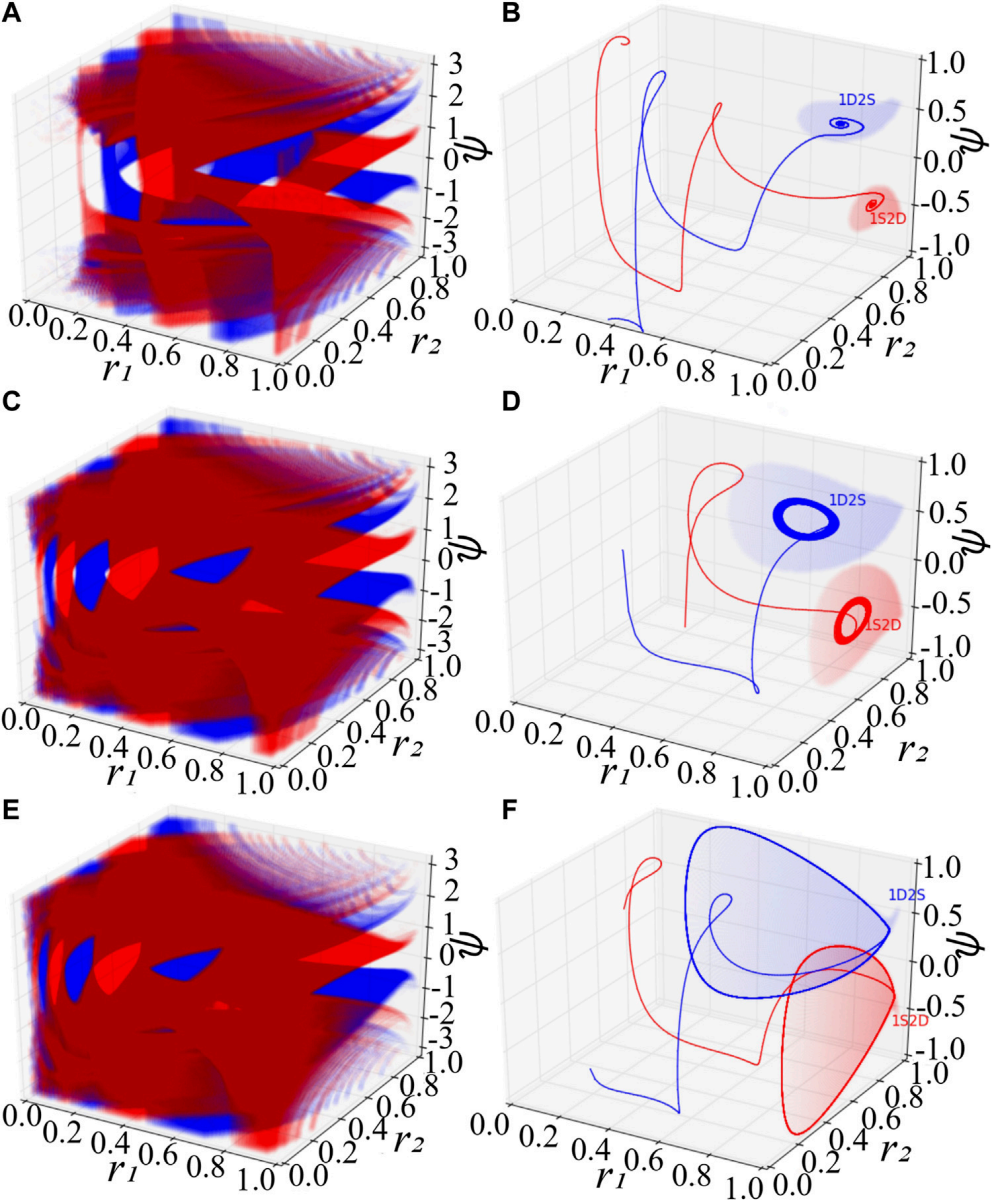
Basins of attraction of chimera states for the low-dimensional system Eq. 7. **(A,B)** The stable chimera states. **(C,D)** The breathing chimera states. **(E,F)** The long-period breathing chimera states. In **(A)**, **(C)**, and **(E)**, the red and blue color denote respectively the basins of attraction of 1S2D and 1D2S. The notations 1S2D represent that the first population is synchronized and the second is desynchronized, and 1D2S denote that the first population is desynchronized and the second is synchronized. Mathematically, 1S2D with *r*_1_ = 1 and *r*_2_ < 1, and 1D2S with *r*_1_ < 1 and *r*_2_ = 1. In **(B)**, **(D)** and **(F)**, red and blue solid lines are trajectories with random initial conditions inside the red and blue attracting basins respectively. Red and blue areas in **(B)**, **(D)** and **(F)** show the special attracting basins with the initial conditions *r*_1_ = 1 and *r*_2_ = 1 respectively. For the simulation, we set *β* = 0.1, *A* = 0.20 in **(A,B)**, *A* = 0.28 in **(C,D)**, and *A* = 0.35 in **(E,F)**.

System Eq. 7 consists of the order parameter *r*_1_ of the first population, that of the second one, and the mean-phase difference *ψ*. With special initial conditions, one could observe remarkable phenomena (Abrams et al., 2008), where the first (second) population is synchronized with *r*_1_ = 1 (*r*_2_ = 1) and the second (first) is desynchronized with *r*_2_ < 1 (*r*_1_ < 1). For convenience, we denote these two kinds of chimera states by 1S2D and 1D2S, correspondingly. Figures 1A,C,E exhibit scatter plots of the basins of attraction of chimera states colored in red (blue), a set of initial conditions leading the low-dimensional system Eq. 7 to approaching 1S2D (1D2S). At each initial value of *r*_1_, *r*_2_ and *ψ*, we independently integrate Eq. 7 long enough, so that the distribution of the state of the oscillators becomes stationary. As predicted by the stability diagram of chimera states (Abrams et al., 2008), stable chimera corresponds to a point, breathing chimera show as a stable limit cycle. The colored region in Figures 1A,C,E indicates, respectively, the basin of attraction of stable, breathing and long-period breathing chimera states. The colored regions of Figures 1B,D,F show the basin of attraction especially with initial value *r*_1_ = 1 for 1S2D and *r*_2_ = 1 for 1D2S. Solid lines in red and blue are trajectories with random initial conditions within the red and blue basins, and they will approach 1S2D and 1D2S respectively. Here, we fix the phase shift *β* = 0.1 and set the coupling disparity *A* = 0.20 in Figures 1A,B, *A* = 0.28 in Figures 1C,D and *A* = 0.35 in Figures 1E,F.

**FIGURE 2:**
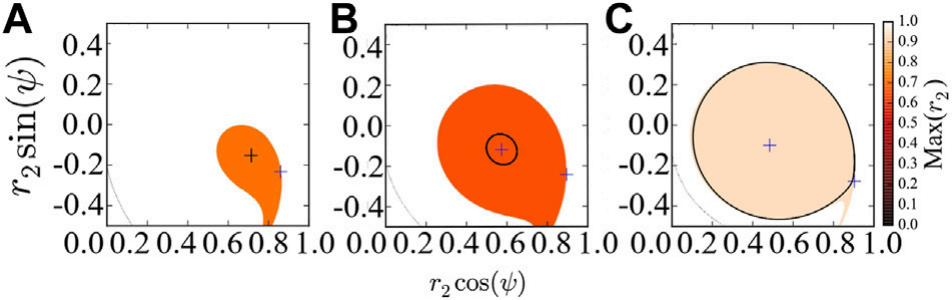
Basins of attraction of chimera states of the reduced system Eq. 7, which are colored with respect to the maximum value *Max*(*r*_2_). **(A)** The system converges to the stable chimera (denoted by black plus). **(B)** The system converges to the breathing chimera (denoted by black circle). **(C)** The system converges to the long-period breathing chimera (denoted by black circle). In **(A–C)**, we regard *r*_2_ and *ψ* as polar coordinates, and the blue pluses indicate the location of fixed points. For the simulation, the initial values (*r*_2_, *ψ*) = (0.7, − 0.1) are inside the basin of attraction of chimera states, and we fix *β* = 0.1 and vary *A* = 0.20 **(A)**, *A* = 0.28 **(B)** and *A* = 0.35 **(C)**.

Figure 2 provides the basins of attraction of the stable chimera in (a), of the breathing chimera in (b), and of the long-period breathing chimera in (c), *via* perturbing the second population *r*_2_ and keeping the first population synchronized, i.e., *r*_1_ = 1. As predicted by the stability diagram of chimera states (Abrams et al., 2008), the red region in Figures 2A–C indicates, respectively, the basin of attraction of stable, breathing and long-period breathing chimera states with *Max*(*r*_2_) < 1. This system Eq. 7 always has a fixed point at *r*_2_ = 1 and *ψ* = 0, and its corresponding attracting basin is colored in white with *Max*(*r*_2_) = 1. Here, for simplicity, we use the maximum value of *r*_2_ denoted by *Max*(*r*_2_) to approximate the basins of attraction of the different states. Saddles colored in blue are always located at the basin boundary.

The linear stability diagram for chimera states was identified by a bifurcation analysis (Abrams et al., 2008), but it does not show how stable a chimera state is under large perturbations. Moreover, in realistic situations, a certain degree of perturbations are largely unavoidable and may drive the system from one desirable state to other unpredictable states. Therefore, it is crucial to investigate its stability against even large perturbations.

Menck et al. (2013) proposed the concept of basin stability (BS) , which is related to the volume of the basin of attraction of a desirable state and quantifies the likelihood that the system returns back to the previous state or converges to an appropriate state after being subjected to even large perturbations. Provided that random perturbations on the system correspond to a uniform distribution on the parameter space, BS of chimera states is equal to the percentage of the volume of their corresponding attracting basins.

**FIGURE 3:**
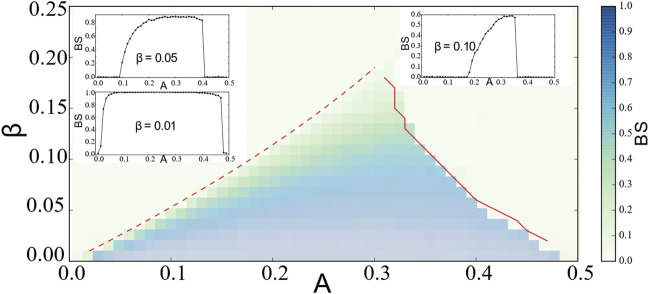
The projection of basin stability (BS) of chimera states of the reduced system Eq. 7 on the stability diagram *A* and *β*. At each pair of parameters (*A*, *β*), BS is calculated independently with 1000 different realizations. Chimera states exist within the region between the saddle-node curve (the red dashed line) and the homoclinic curve (the red solid line). BS first increases with the increase of *A* and starts decreasing near the homoclinic line as shown in the insets with different values of *β*. The saddle-node curve is approximated by *A*_SN_(*β*) in (Abrams et al., 2008). The homoclinic curve is approximated numerically. For further information, we recommend the analysis of the stability diagram in reference Martens et al. (2016).

Numerically, we perturb the three parameters (*r*_1_, *r*_2_, *ψ*) *M* times independently inside the range of [0, 1] × [0, 1] × [ − *π*, *π*], count the number denoted by *S* of the system retaining back to chimera states, and then approximate the basin stability BS as the likelihood $\frac{S}{M}$ . As shown in Figure 3, the shaded area denotes the basin stability of chimera states, the basin stability is projected on the parameter space regarding *A* and *β*. They are detectable between the two red bifurcation lines with BS>0 . The diameter of the attracting basin BS of chimera states increases as *A* increases, and therefore their BS increases. With further increases in *A*, BS starts decreasing slightly. As the amplitude of *β* expands and touches the saddle at the homoclinic bifurcation, chimera states therein vanish leading to BS=0 . With the variations of *A* and *β*, the values of BS are not always equal to 1, indicating that there are coherent and incoherent states coexisting with chimera sates.

## 4 BS on networks

**FIGURE 4:**
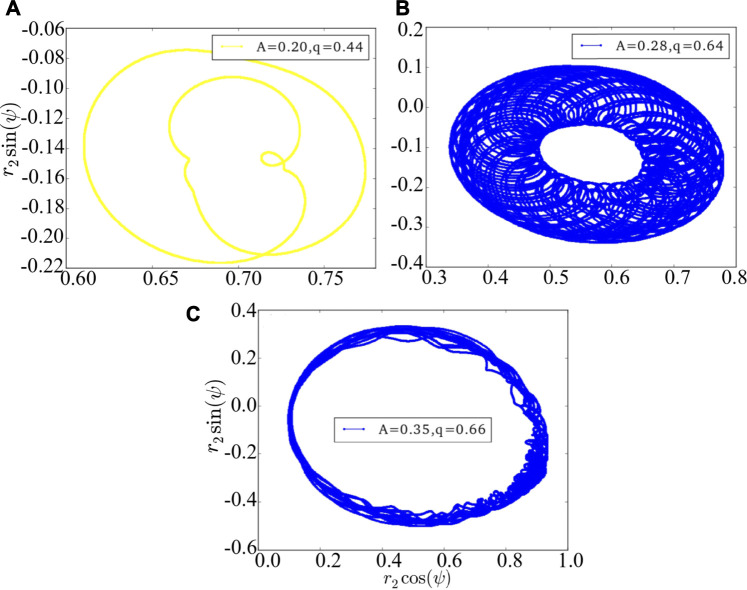
The phase portrait of the original dynamics (1). **(A)** is periodic chimera, and **(B,C)** are quasi-periodic chimeras. The realizations vary depending crucially on the initial conditions, perturbations and parameter values. Here we use arbitrary values of parameters *A* = 0.20 and *q* = 0.44 in **(A)**, *A* = 0.28 and *q* = 0.64 in **(B)**, and *A* = 0.35 and *q* = 0.66 in **(C)**.

The above results are achieved under the Ott-Antonsen ansatz by considering a restricted class of density functions following the form of a Poisson kernel. Next, we analyze the original dynamics (1) and investigate its basin stability, to compare with the three cases of Figure 1 as observed from the low-dimensional system Eq. 7. Initially, the system is in the state of stable chimera, breathing chimera or long-period breathing chimera as predicted by the low-dimensional solution. Numerically, we observe that the system after perturbations will probably converge to periodic chimera instead of stable chimera with *A* = 0.20, quasi-periodic chimera instead of breathing chimera with *A* = 0.28, quasi-periodic chimera instead of long-period breathing chimera with *A* = 0.35. With the variations of *r*_1_, *r*_2_ and *ψ*, trajectories of periodic chimera are stable and periodic with a closed curve in polar coordinates, and trajectories of quasi-periodic chimera are periodic while not stable with many closed curves. We show different realizations of periodic chimera in Figure 4A and quasi-periodic chimeras Figures 4B,C after perturbations with different parameter values. As shown in Figure 4, with periodic chimera suggests that the trajectories of *r*_1_, *r*_2_ and *ψ* are stable and periodic, and the trajectories are a closed curve in polar coordinates.

In comparison with the results of the reduced system, in what follows, we provide the results *via* perturbing especially the second population.

Chimera states require carefully selected initial conditions, and, therefore, it is interesting to quantify BS of chimera states *via* perturbation of the fraction *q* ∈ [0, 1] of nodes in both populations. In comparison with the results of the reduced system, the system is initially located in one of stable chimera, breathing chimera or long-period breathing chimera as predicted by the low-dimensional solution. We select at random *q* percent nodes in two populations, randomly draw initial values *θ* of the selected nodes from [0, 2*π*], and launch the system to reach stationary states *S*. At each q, we repeat the above process *M*_*q*_ times independently, count the number *N*_*S*;*q*_ of reaching *S*, and quantify basin stability of *S* *via*

$$BS\left(S;q\right)=\frac{N_{S;q}}{M_{q}},$$ (8)

with *M*_*q*_ = *∑*_*S*_ *N*_*S*;*q*_.

Numerically, we observe that the degree of phase coherence becomes periodic or quasi-periodic after even large perturbations. In Figure 4A, with the coupling disparity *A* = 0.20, the order parameter *r*_1_ of the first population or *r*_2_ of the second population starts oscillating and the stable chimera states become periodic. With *A* = 0.28 in Figure 4B or 0.35 in Figure 4C, *r*_1_ or *r*_2_ displays irregular periodicity and breathing chimeras become quasi-periodic. Therefore, phase portraits can not be depicted solely by the Ott-Antonsen ansatz, though this does work for the case of the low-dimensional system. Realizations of periodic and quasi-periodic chimera states of Eq. 7 vary and depend heavily on the initial conditions and parameter values.

**FIGURE 5:**
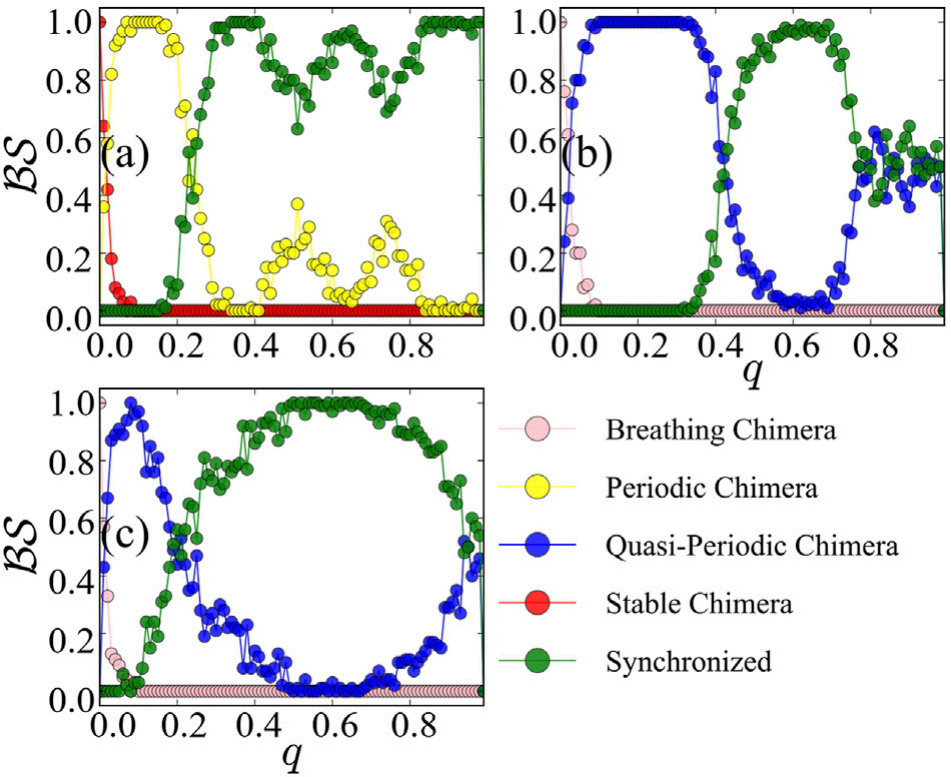
Basin stability BS of different states of the original system, with respect to the percentage *q* of the perturbed nodes of two populations. The coupling disparity is set by *A* = 0.20 in **(A)**, *A* = 0.28 in **(B)**, and *A* = 0.35 in **(C)**. The emerged states include breathing chimera (in pink), periodic chimera (in yellow), quasi-periodic chimera (in blue), stable chimera (in red), and synchronized (in green). Initially, the system is in the region of stable chimera **(A)** and breathing chimera **(B,C)** as predicted by the low-dimensional model. For the simulation, we set *β* = 0.1, *M*_*q*_ = 100 and *N*_*σ*_ = 128.

In Figure 5, we plot the basin stability BS of different states as a function of *q*. A synchronized state always exists, and we hereafter focus on chimera states. BS of stable chimera in Figure 5A [resp. breathing chimera in Figures 5B,C] decreases very fast with a slight increase of *q*, and it vanishes at a small percentage *q* ≈ 0.1. Afterwards, periodic chimera in Figure 5A or quasi-periodic in Figures 5B,C become dominant. With increasing *q*, BS of periodic chimera and quasi-periodic chimera decreases. In Figure 5A, the basins of attraction of chimera states are typically smaller than that of the synchronized state with randomly selected initial perturbations. Interestingly, with further increases in *q*, BS of periodic chimera exhibits a *bimodal* curve with two peaks, respectively, at *q* ≈ 0.5 and *q* ≈ 0.7. BS of periodic chimera states vanishes at *q* ≈ 1. We also observe such a bimodal feature of basin stability of multichimera states in the coupled FitzHugh-Nagumo model. In Figures 5B,C, with further increases in *q*, BS of quasi-periodic chimera increases again and then persists at 0.5. Interestingly, we observe that, in a certain region of *q*, *BS* is higher than that when only the second population is perturbed.

**FIGURE 6:**
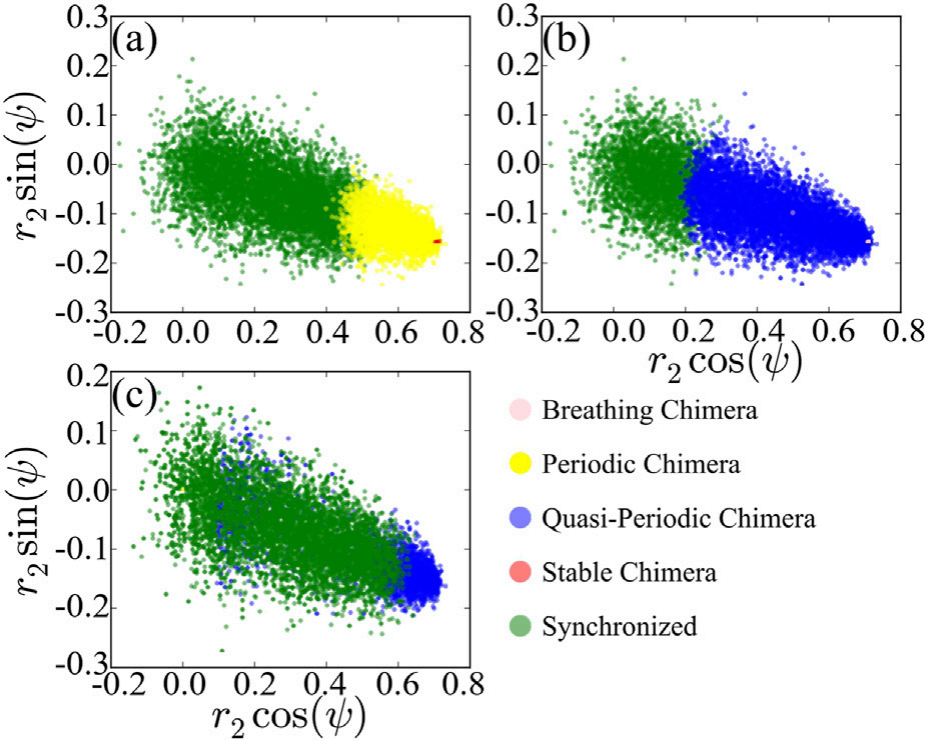
Projection of basins of attraction of different states of the original system, with *A* = 0.20 **(A)**, *A* = 0.28 **(B)** and *A* = 0.35 **(C)**. For comparing the results to Figure 1, we regard *r*_2_ and *ψ* as polar coordinates.

To compare with the solution of the low-dimensional model Eq. 7, we record values of polar coordinates *r*_2_ and *ψ* with the initial value *r*_1_ = 1 as basin of attraction of the corresponding stationary state with respect to *A* and then project basins of attractions of different states on the space regarding the polar coordinates as shown in Figure 6. We observe that dominant chimeras are periodic in Figure 6A rather than stable or quasi-periodic in Figures 6B,C instead of breathing chimera. The basin boundary between different states is not clearly separated, in contrast to that shown in Figure 1 of the reduced system Eq. 7. Moreover, the basins of attraction between different states are overlapped. Recall that the calculation of BS of chimera states on the low-dimensional model is based on the equivalence of the uniform distribution of the space to random perturbations on chimera states on networks. Conversely, as shown in Figure 6, scatter plots of the attracting basin of chimera states regarding *r*_2_ sin(*ψ*) and *r*_2_ cos(*ψ*) are centralized rather than uniformly distributed in the space.

For a comparison between the reduced system and the original system, in terms of basin stability, the dominated states in the reduced system are stable and are breathing chimera states, with small basins of attraction. However, the original system is dominated by periodic and quasi-periodic chimera states, in contrast to the model after reduction. The original system being subjected to even more and large perturbations, the curve of basin stability of the chimera states behaves bimodally. Therefore, the low-dimensional system under the Ott-Antonsen ansatz cannot capture the behavior of the basins.

## 5 Chimera states on brain networks

Up to now, we have investigated the basins of attraction of chimera states in original and reduced Kuramoto networks. In this section, we focus the influence of stimulating regions on the chimera states on brain networks.

Firstly, we use the diffusion imaging data to generate brain networks. The Diffusion imaging data are available from the Human Connectome Project (HCP), WU-Minn Consortium (https://www.humanconnectome.org). HCP recruits subjects in the age range of 22–35 years, and subjects are scanned on a customized Siemens 3 T “Connectome Skyra” at Washington University, using a standard 32-channel Siemens receive head coil and a “body” transmission coil (Van Essen et al., 2013). For the simplification, we take the diffusion pre-processed data of 30 individual participant scans, randomly selected, and use DSI Studio (http://dsi-studio.labsolver.org/) to perform whole-brain fiber tractography between brain regions. To obtain the fiber connectivity matrix of each participant, the fiber threshold is set by 0.001, as the default value of DSI Studio, to filter out a small number of connecting tracks. In this case, the fiber connectivity between brain regions smaller than the threshold will be ignored in the connectivity matrix (Yeh, 2017). We use the automated anatomical atlas (AAL2), with 94 cortical brain regions (Rolls et al., 2015), and further obtain the fiber connectivity matrices for these participants, accounting for fiber numbers between brain regions. The connectivity matrix and graph theoretical analysis are conducted by using DSI Studio (http://dsi-studio.labsolver.org). To minimize the impact of bias in the tractography parameter scheme on connectivity matrix generation, we use the averaged fiber connectivity matrix, across 30 subjects, to simulate the brain networks.

Based on the generated networks, we use the WCOs Eq. 2, employ the stochastic Euler-Maruyama method with time step size 0.001 s, and set the initial conditions *E*_*i*_(0) = 0.1, *I*_*i*_(0) = 0.1 with *i* = 1, …, *N*. The connection strength *A*_*ij*_ in Eq. 2 is obtained by normalizing the averaged fiber connectivity matrix, i.e., *A*_*ij*_ = *n*_*ij*_/*n*_*s*_, where *n*_*ij*_ is the fiber count between regions *i* and *j*, and *n*_*s*_ is the sum of fiber count in the whole brain. The dynamical perturbation of the stimulated region *P*_*i*_ = 1.15 is utilized as a single regional stimulation. After stimulation, signals propagate through the network connectivity from the stimulated regions and others, (Bansal et al., 2019).

**FIGURE 7:**
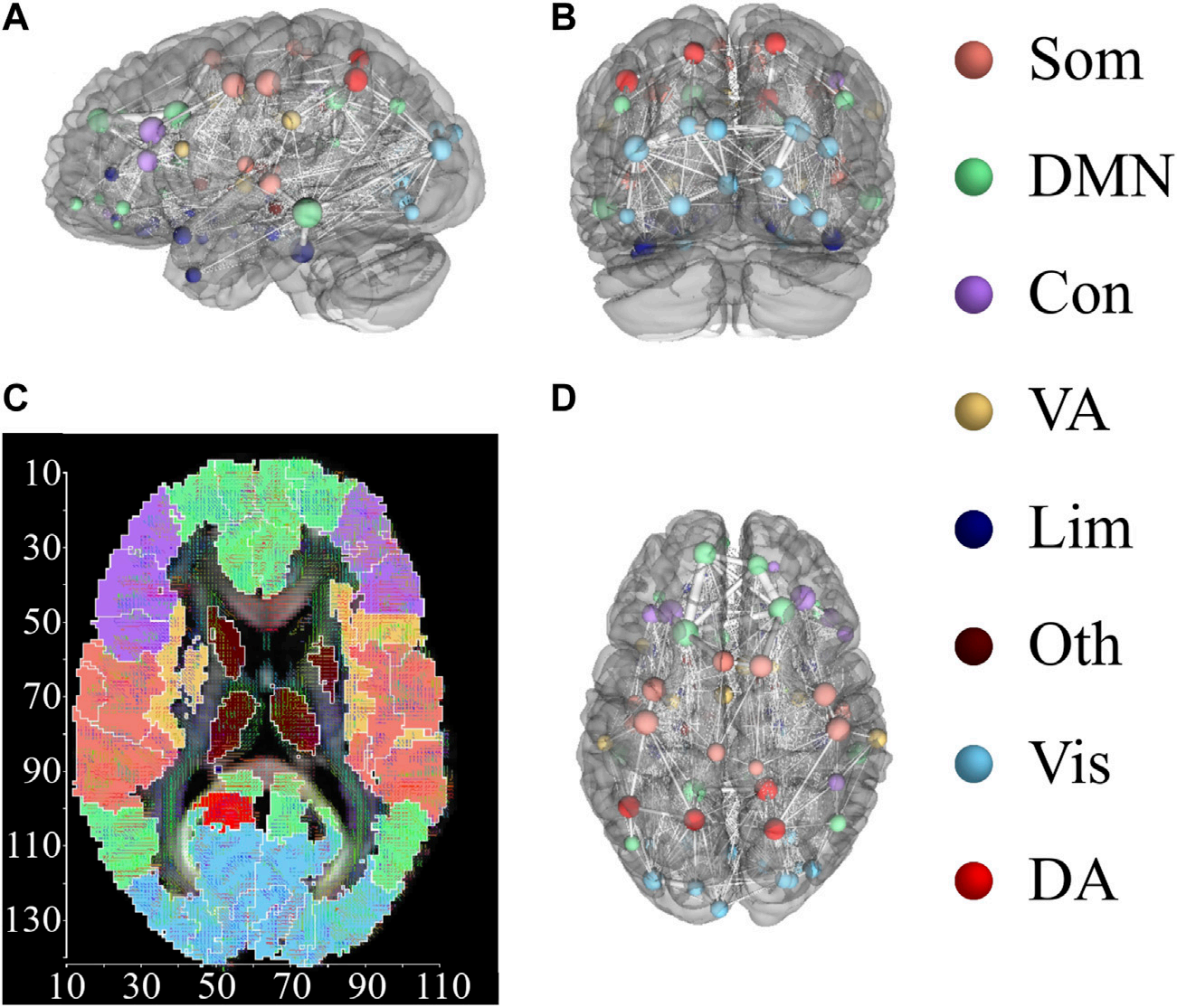
Distribution of 94 AAL2 brain regions within 8 cognitive systems. **(A,B)** and **(D)** show 3-dimensional spatial distribution of the 94 brain regions in 3 directional views, with left view **(A)**, back view **(B)**, and top view **(D)**. Brain regions with the same color belong to the same cognitive systems. The connections between brain regions are displayed, with the thickness representing the connection strength. **(C)** Spatial mappings show the distribution of 8 cognitive systems. The color of map is connected to the cognitive system shown in the right column. This figure is drawn by using DSI Studio.

In what follows, we focus on the investigation of the impact on brain dynamics (stimulated region) and the quantification of the induced chimera states from different regions. To characterize the emerged states in brain dynamics, we focus on synchronized patterns on brain systems, where regions are divided into different functional regions with similar cognitive processes. For this network, each region is assigned to one of eight cognitive systems (Yeo et al., 2011), including somatomotor (Som), default mode network (DMF), control (Con), dorsal attention (DA), limbic (Lim), visual (Vis), ventral attention (VA), other (Oth, subcortical regions could not be assigned to any system) (Dimulescu et al., 2021). Figure 7 exhibits the assignment of 94 AAL2 brain regions within eight cognitive systems. Figures 7A–C shows the three dimensional distribution of 94 AAL2 brain regions within brain, with same color nodes assigned to one cognitive system. Figure 7D is the spatial mappings of 8 cognitive systems.

To quantify the degree of synchronization between brain regions, we use the order parameter based on a single regional stimulation. For the *N* coupling WCOs, the phase of the *i*-th node follows

$$\theta_{i}\left(t\right)=\arctan\frac{I_{i}\left(t\right)}{E_{i}\left(t\right)},\;\;i=1,2,\ldots ,N.$$ (9)

The order parameter averaged across a long period of time *T* indicates the global synchrony, i.e.,

$$r_{N}={\left\langle r_{\sigma }\left(t\right)\right\rangle }_{T}.$$ (10)

where *σ* represents all regions, i.e., *N*_*σ*_ = *N*. For instance, if all regions move coherently and act like a giant component, *r*_*N*_ ≈ 1, otherwise, *r*_*N*_ ≈ 0 (Strogatz, 2000). In numerical simulations, we set *T* = 1 s to estimate the averaged order parameter.

Additionally, we use the order parameter to investigate the synchronized activities between pairs of cognitive systems based on a regional stimulation. In particular, for each pair of systems *ξ*_*i*_ and *ξ*_*j*_ with $N_{\xi_{i}}$ and $N_{\xi_{j}}$ regions, respectively, the phase synchronization between *ξ*_*i*_ and *ξ*_*j*_ at time *t* follows

$$r_{\xi_{i},\xi_{j}}\left(t\right){\text{e}}^{\text{i}\Phi \left(t\right)}=\frac{1}{N_{\xi_{i}}+N_{\xi_{j}}}\underset{k\in \left(\xi_{i}\cup \xi_{j}\right)}{\sum }{\text{e}}^{\text{i}\theta_{k}\left(t\right)},$$ (11)

where Φ(*t*) is the averaged phase of oscillators within cognitive systems *ξ*_*i*_ and *ξ*_*j*_. The cognitive system-level order parameter is calculated by averaging $r_{\xi_{i},\xi_{j}}\left(t\right)$ on a long period *T* (Bansal et al., 2019), as

$$r_{\xi_{i},\xi_{j}}={\left\langle r_{\xi_{i},\xi_{j}}\left(t\right)\right\rangle }_{T},$$ (12)

for each pair of cognitive systems, i.e., *i*, *j* = 1, 2, …, 8. For a given regional stimulation, the system Eq. 2 may exhibit different final states based on different coupling strengths *c*_5_. To identify the synchronization patterns, we define a synchronization threshold *r*_Th_, such that two cognitive systems *ξ*_*i*_ and *ξ*_*j*_ are considered to be synchronized if $r_{\xi_{i},\xi_{j}}\geq r_{\text{Th}}$ . In simulations, we set *r*_Th_ = 0.8 (Bansal et al., 2019).

**FIGURE 8:**
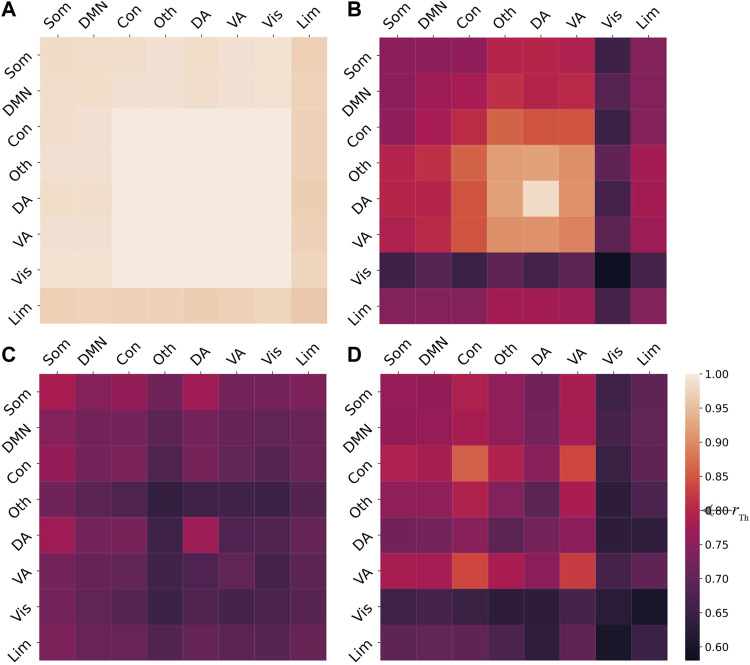
**(A)** Coherent state generated with the coupling strength *c*_5_ = 1000. **(B)** Chimera state with the coupling strength *c*_5_ = 330. **(C)** Metastable state with the coupling strength *c*_5_ = 200. In **(A–C)**, we stimulate the 1-st brain region by applying a constant external input, i.e., *P*_*i*_ = 1.15 if *i* = 1 and *P*_*i*_ = 0 otherwise. **(D)** Chimera state of the brain dynamics without stimulation for *c*_5_ = 330. The order parameter $r_{\xi_{i},\xi_{j}}$ and a threshold *r*_Th_ = 0.8 are used to classify the final states of the brain dynamics. The order parameter threshold *r*_Th_ is marked on the colorbar. To obtain a stationary state, we integrate the brain dynamics Eq. 2 in time interval [0, 150 s] with step size = 0.001 s, and set the initial conditions *E*_*i*_(0) = 0.1, *I*_*i*_(0) = 0.1 with *i* = 1, …, *N*.

To explore how a single region drives the brain dynamics with different underlying couplings, based on varying the coupling strength *c*_5_, we simulate the dynamics Eq. 2 and obtain three different synchronization patterns, including (i) the coherent state, (ii) the chimera state with coexisting synchronized and desynchronized subpopulations, and (iii) the metastable state with the absence of any large-scale stable synchronized oscillations (Bansal et al., 2019). Figure 8 shows the corresponding numerical results, with (a) the coherent state with the order parameter $r_{\xi_{i},\xi_{j}}\geq r_{\text{Th}}$ and (b) the chimera state with coexisting of synchronized and desynchronized systems. The synchronization level within or between ventral attention, other, dorsal attention, is higher than other systems, i.e. the order parameters $r_{\xi_{i},\xi_{j}}$ within or between these three cognitive systems have larger values. Besides, dorsal attention is the most synchronized cognitive system with the largest order parameter $r_{\xi_{i},\xi_{j}}$ . Synchronization of visual between other cognitive systems (including itself) has a lower value of $r_{\xi_{i},\xi_{j}}<r_{\text{Th}}$ . Especially, the order parameter $r_{\xi_{i},\xi_{j}}$ within visual is the smallest. Figure 8C illustrates the metastable state with the order parameter $r_{\xi_{i},\xi_{j}}<r_{\text{Th}}$ for any *i*, *j* = 1, 2, …, 8. To compare the influences of chimera state after stimulation, we set *P*_*i*_ = 0 for *i* = 1, 2, …, *N*. Figure 8D shows that the brain dynamics exhibit a chimera state without stimulation, with the smaller order parameters $r_{\xi_{i},\xi_{j}}$ compared to (b). Therefore, the chimera state of the brain dynamics without stimulation has the lower synchronization level between cognitive systems compared to the single regional stimulation.

We further focus on how stimulation on brain regions impacts the induced chimera states of the model Eq. 2. Given the coupling strength *c*_5_ = 330, we stimulate the *i*-th brain region and integrate Eq. 2 10 times for each region *i*, with *i* ranging from 1 to *N*. For each simulation, the system will converge to either coherent, chimera, or metastable states. To identify the final state, we calculate the system-level order parameter matrices with elements $r_{\xi_{i},\xi_{j}}$ . Each of the resulted matrices is binarized *via* a matrix *B*, i.e., $B_{\xi_{i},\xi_{j}}$ = 1 if *ξ*_*i*_ and *ξ*_*j*_ are synchronized $\left(r_{\xi_{i},\xi_{j}}\geq r_{\text{Th}}\right)$ , otherwise $B_{\xi_{i},\xi_{j}}$ = 0. The coherent state is identified if all elements $B_{\xi_{i},\xi_{j}}$ of the binarized matrix *B* are 1; metastable state has the binarized matrix *B* with each element $B_{\xi_{i},\xi_{j}}$ equal to 0. We additionally classify the final state by the probability of $r_{\xi_{i},\xi_{j}}\geq r_{\text{Th}}$ in the binarized matrix *B*, denoted by $P=\left(\sum_{i,\;j=1}^{8}B_{\xi_{i},\xi_{j}}\right)/64$ . The coherent state is with *P* = 1, chimera state with 0 < *P* < 1, and the metastable state with *P* = 0.

**FIGURE 9:**
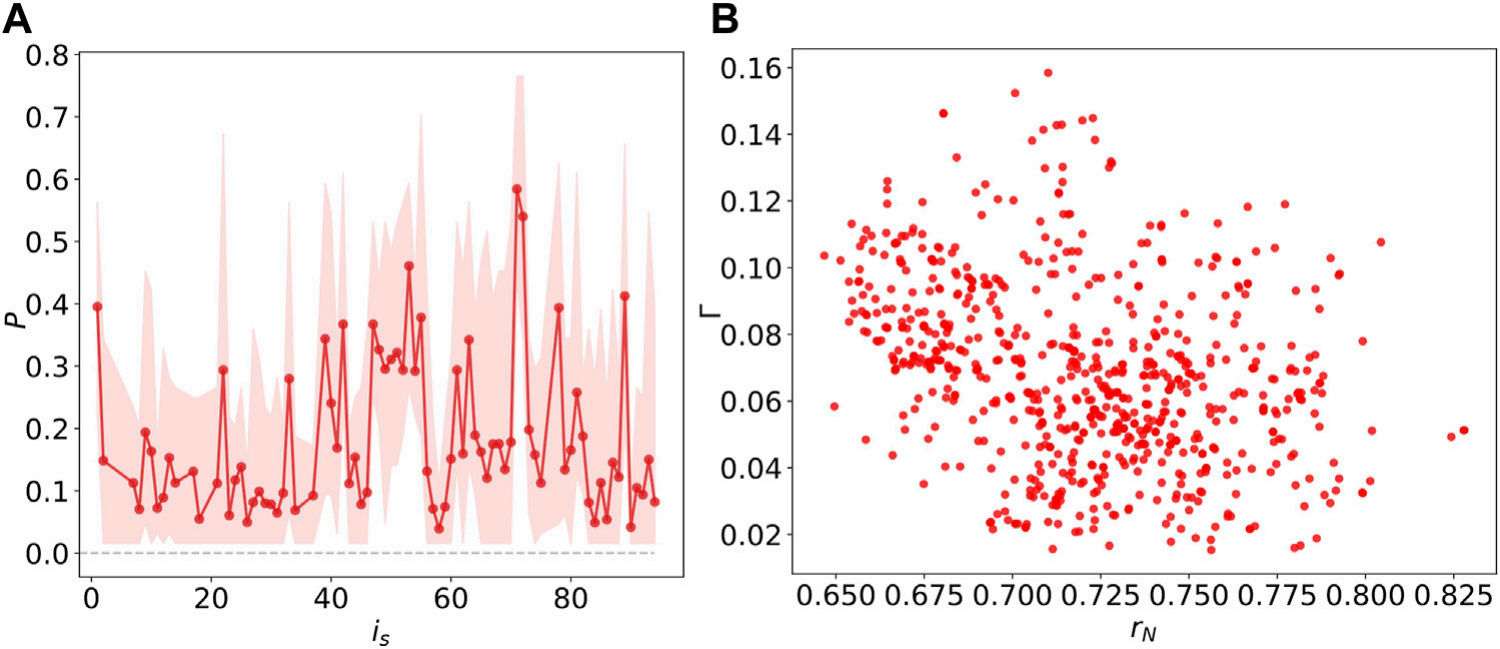
**(A)** The probability *P* of $r_{\xi_{i},\xi_{j}}\geq r_{\text{Th}}$ in the order parameter matrix versus the stimulated node index *i*_*s*_. The red line represents the variations of average probability versus the stimulated node index. Each red dot corresponds to a stimulated region. The shaded area shows the variation range of the probability. The dashed line helps distinguish the probability *P* > 0 in the shaded area. **(B)** The chimera index Γ is weakly and negatively correlated with *r*_*N*_, with the correlation coefficient *r* ≈ − 0.33 and the *p*-value *p* ≈ 2.87 × 10^−19^. One red dot describes a chimera state generated *via* the single regional stimulation. For each single regional stimulation, simulating Eq. 2 ten times, using 0 < *P* < 1 identifies the generated chimera states, and *P*_*i*_ = 1.15. The time interval of simulating Eq. 2 is [0, 150 s], with step size = 0.001 s. The initial conditions are *E*_*i*_(0) = 0.1, *I*_*i*_(0) = 0.1 with *i* = 1, …, *N*.

We stimulate each region *i* and integrate Eq. 2 10 times for each *i*, with *N* = 94. For the identified chimera state based on *P*, we calculate the index of the stimulated region *i*_*s*_ and the corresponding number of simulation $n_{i_{s}}$ , where 1 ≤ *i*_*s*_ ≤ 94 and $1\leq n_{i_{s}}\leq 10$ . There are 731 chimera states, found with different stimulated region *i*_*s*_ and the number of simulation $n_{i_{s}}$ . For a fixed stimulated region *i*_*s*_ with $n_{i_{s}}$ chimera states, we calculate the average probability, maximal probability, minimal probability, and exhibit the variations of these probabilities versus stimulated node index *i*_*s*_ in Figure 9A. As shown in Figure 9A, all the probabilities satisfy 0 < *P* < 1, indicating chimera states with coexisting of synchronization and desynchronization.

As described in (Shanahan, 2010; Bansal et al., 2019), an ideal chimera state occurs with half of the population synchronized and the others desynchronized. After identifying the chimera states with different stimulated node *i*_*s*_ and the corresponding number of simulation $n_{i_{s}}$ , we analyze numerical results by using two measures of synchronization, consisting of the Kuramoto order parameter and the chimera index. The classical Kuramoto order parameter is calculated *via* Eq. 10, capturing the level of synchrony (Kuramoto, 1975; Strogatz, 2000). The chimera index describes how close a final state to an ideal chimera state (Shanahan, 2010; Hizanidis et al., 2016; Bansal et al., 2019). For a brain network with *M* cognitive systems *ξ*_1_, *ξ*_2_, …, *ξ*_*M*_, the chimera index follows

$$\Gamma =\frac{{\left\langle \gamma_{\text{ch}}\left(t\right)\right\rangle }_{T}}{\Gamma_{\text{max}}},$$ (13)

where

$$\gamma_{\text{ch}}\left(t\right)=\frac{1}{M-1}\overset{M}{\underset{i=1}{\sum }}{\left(r_{\xi_{i}}\left(t\right)-{\left\langle r_{\xi }\left(t\right)\right\rangle }_{M}\right)}^{2}.$$ (14)

The chimera index Γ is averaged over the long time *T*, representing the averaged diversities in the order parameters within the *M* cognitive systems (Bansal et al., 2019). The normalization factor Γ_max_ = 5/36 depicts the maximal variations of the order parameter corresponding to an ideal chimera state (Shanahan, 2010; Bansal et al., 2019). The instantaneous quantity ${\left\langle r_{\xi }\left(t\right)\right\rangle }_{M}=\left(\sum_{i=1}^{M}r_{\xi_{i}}\left(t\right)\right)/M$ evaluates the averaged synchronization of *M* cognitive systems at time *t*. Figure 9B illustrates the chimera index Γ is weakly and negatively correlates with the Kuramoto order parameter *r*_*N*_. This implies that stimulating the node inducing an ideal chimera state tends to have a lower global synchrony.

**FIGURE 10:**
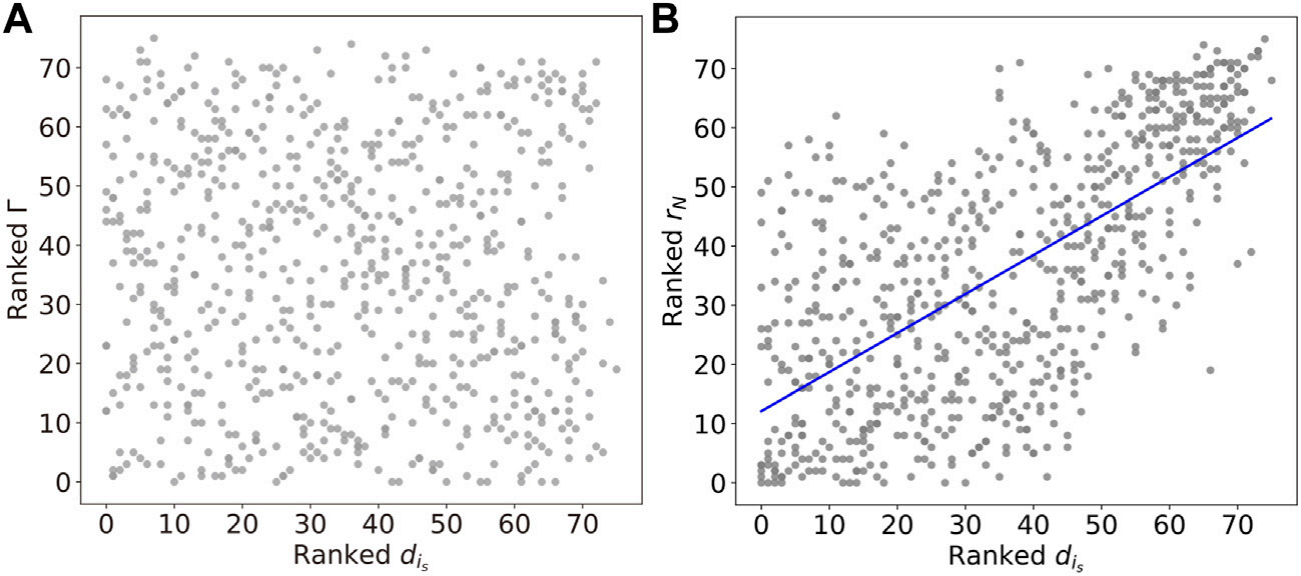
**(A)** The linear relations between the Kuramoto order parameter *r*_*N*_ and degree $d_{i_{s}}$ , with the correlation coefficient *r* ≈ 0.66 and the *p*-value *p* ≈ 2.55 × 10^−94^. The blue line represents the best-fit line. **(B)** The chimera index Γ shows no apparent correlation with $d_{i_{s}}$ .

To illustrate further how the chimera states are constrained by the underlying network structure, we investigate the Kuramoto order parameter and the chimera index, as a function of the degree $d_{i_{s}}=\sum_{j=1}^{N}A_{i_{s},j}$ . Figure 10A shows a linear relation between the Kuramoto order parameter *r*_*N*_ and the stimulated-nodes’ degree $d_{i_{s}}$ . Results show that nodes *i*_*s*_ with higher degree tend to produce a global synchronized state. This suggests that the synchronized state induced by high-degree node is more robust with the presence of noise. However, as shown in Figure 10B, the ranked chimera index Γ has no such clear correlation with the ranked degree $d_{i_{s}}$ .

## 6 Conclusion

In the paper, we have firstly investigated the basin of attraction of stable and breathing chimera states of the solvable model (Abrams et al., 2008) with and without the Ott-Antonsen ansatz, and we have implemented basin stability on chimera states and quantified their stability after even large perturbations. Quantitatively, we have shown that periodic and quasi-periodic chimera states of networked oscillators, instead of stable and breathing chimera states in the reduced system, dominate the desynchronized states of the full system. Interestingly, we have observed that the curve of basin stability of chimera states becomes bimodal. The same process could be widely implemented on where chimera states are observed. It would also be worth looking at experiments for future work.

Additionally, we have also employed a biologically motivated, networked model WCOs, to investigate how the induced chimera states are influenced by the stimulation of various regions. Stimulating single region with different coupling strength could potentially force the system to three states, consisting of the coherent, chimera, and metastable state. For the chimera behavior on brain networked model, the chimera state without stimulation exhibits low synchronization between cognitive systems. Besides, the variations of Kuramoto order parameter suggest that higher-degree nodes could induce higher synchronization influences compared to the lower ones.

## Data Availability

The raw data supporting the conclusions of this article will be made available by the authors, without undue reservation.

## References

[B1] Abrams D. M. Mirollo R. Strogatz S. H. Wiley D. A. (2008). Solvable model for chimera states of coupled oscillators. Phys. Rev. Lett. 101, 084103. 10.1103/PhysRevLett.101.084103 18764617

[B2] Abrams D. M. Strogatz S. H. (2004). Chimera states for coupled oscillators. Phys. Rev. Lett. 93, 174102. 10.1103/PhysRevLett.93.174102 15525081

[B3] Arenas A. Díaz-Guilera A. Kurths J. Moreno Y. Zhou C. (2008). Synchronization in complex networks. Phys. Rep. 469, 93–153. 10.1016/j.physrep.2008.09.002

[B4] Bansal K. Garcia J. O. Tompson S. H. Verstynen T. Vettel J. M. Muldoon S. F. (2019). Cognitive chimera states in human brain networks. Sci. Adv. 5, eaau8535. 10.1126/sciadv.aau8535 30949576 PMC6447382

[B5] Bansal K. Medaglia J. D. Bassett D. S. Vettel J. M. Muldoon S. F. (2018). Data-driven brain network models differentiate variability across language tasks. PLoS Comput. Biol. 14, e1006487. 10.1371/journal.pcbi.1006487 30332401 PMC6192563

[B6] Chouzouris T. Omelchenko I. Zakharova A. Hlinka J. Jiruska P. Schöll E. (2018). Chimera states in brain networks: Empirical neural vs. modular fractal connectivity. Chaos 28, 045112. 10.1063/1.5009812 31906648

[B7] Dimulescu C. Gareayaghi S. Kamp F. Fromm S. Obermayer K. Metzner C. (2021). Structural differences between healthy subjects and patients with schizophrenia or schizoaffective disorder: A graph and control theoretical perspective. Front. Psychiatry 12, 991. 10.3389/fpsyt.2021.669783 PMC8273511 34262489

[B8] Hagerstrom A. M. Murphy T. E. Roy R. Hövel P. Omelchenko I. Schöll E. (2012). Experimental observation of chimeras in coupled-map lattices. Nat. Phys. 8, 658–661. 10.1038/nphys2372

[B9] Hizanidis J. Kouvaris N. E. Zamora-López G. Díaz-Guilera A. Antonopoulos C. G. (2016). Corrigendum: Chimera-like states in modular neural networks. Sci. Rep. 6, 1–11. 10.1038/srep22314 26796971 PMC4726386

[B10] Huo S. Tian C. Kang L. Liu Z. (2019). Chimera states of neuron networks with adaptive coupling. Nonlinear Dyn. 96, 75–86. 10.1007/s11071-019-04774-4

[B11] Kang L. Tian C. Huo S. Liu Z. (2019). A two-layered brain network model and its chimera state. Sci. Rep. 9, 1–12. 10.1038/s41598-019-50969-5 31591418 PMC6779761

[B12] Kuramoto Y. Battogtokh D. (2002). Coexistence of coherence and incoherence in nonlocally coupled phase oscillators. Nonlinear Phenom. Complex Syst. 5, 380–385.

[B13] Kuramoto Y. (1975). “Self-entrainment of a population of coupled non-linear oscillators,” in International symposium on mathematical problems in theoretical physics (Springer), 420–422.

[B14] Martens E. A. Panaggio M. J. Abrams D. M. (2016). Basins of attraction for chimera states. New J. Phys. 18, 022002. 10.1088/1367-2630/18/2/022002

[B15] Martens E. A. Thutupalli S. Fourrière A. Hallatschek O. (2013). Chimera states in mechanical oscillator networks. Proc. Natl. Acad. Sci. U. S. A. 110, 10563–10567. 10.1073/pnas.1302880110 23759743 PMC3696826

[B16] Menck P. J. Heitzig J. Marwan N. Kurths J. (2013). How basin stability complements the linear-stability paradigm. Nat. Phys. 9, 89–92. 10.1038/nphys2516

[B17] Muldoon S. F. Pasqualetti F. Gu S. Cieslak M. Grafton S. T. Vettel J. M. (2016). Stimulation-based control of dynamic brain networks. PLoS Comput. Biol. 12, e1005076. 10.1371/journal.pcbi.1005076 27611328 PMC5017638

[B18] Nkomo S. Tinsley M. R. Showalter K. (2013). Chimera states in populations of nonlocally coupled chemical oscillators. Phys. Rev. Lett. 110, 244102. 10.1103/PhysRevLett.110.244102 25165927

[B19] Omel’chenko O. (2013). Coherence–incoherence patterns in a ring of non-locally coupled phase oscillators. Nonlinearity 26, 2469–2498. 10.1088/0951-7715/26/9/2469

[B20] Ott E. Antonsen T. M. (2008). Low dimensional behavior of large systems of globally coupled oscillators. Chaos 18, 037113. 10.1063/1.2930766 19045487

[B21] Panaggio M. J. Abrams D. M. (2015). Chimera states: Coexistence of coherence and incoherence in networks of coupled oscillators. Nonlinearity 28, R67–R87. 10.1088/0951-7715/28/3/r67

[B22] Parastesh F. Jafari S. Azarnoush H. Shahriari Z. Wang Z. Boccaletti S. (2021). Chimeras. Phys. Rep. 898, 1–114. 10.1016/j.physrep.2020.10.003

[B23] Pikovsky A. Rosenblum M. (2008). Partially integrable dynamics of hierarchical populations of coupled oscillators. Phys. Rev. Lett. 101, 264103. 10.1103/PhysRevLett.101.264103 19437642

[B24] Rolls E. T. Joliot M. Tzourio-Mazoyer N. (2015). Implementation of a new parcellation of the orbitofrontal cortex in the automated anatomical labeling atlas. Neuroimage 122, 1–5. 10.1016/j.neuroimage.2015.07.075 26241684

[B25] Shanahan M. (2010). Metastable chimera states in community-structured oscillator networks. Chaos 20, 013108. 10.1063/1.3305451 20370263

[B26] Strogatz S. (2000). From kuramoto to crawford: Exploring the onset of synchronization in populations of coupled oscillators. Phys. D. Nonlinear Phenom. 143, 1–20. 10.1016/s0167-2789(00)00094-4

[B27] Tamaki M. Bang J. W. Watanabe T. Sasaki Y. (2016). Night watch in one brain hemisphere during sleep associated with the first-night effect in humans. Curr. Biol. 26, 1190–1194. 10.1016/j.cub.2016.02.063 27112296 PMC4864126

[B28] Tinsley M. R. Nkomo S. Showalter K. (2012). Chimera and phase-cluster states in populations of coupled chemical oscillators. Nat. Phys. 8, 662–665. 10.1038/nphys2371 25165927

[B29] Van Essen D. C. Smith S. M. Barch D. M. Behrens T. E. Yacoub E. Ugurbil K. (2013). The Wu-minn human connectome project: An overview. Neuroimage 80, 62–79. 10.1016/j.neuroimage.2013.05.041 23684880 PMC3724347

[B30] Wang Z. Liu Z. (2020). A brief review of chimera state in empirical brain networks. Front. Physiol. 11, 724. 10.3389/fphys.2020.00724 32714208 PMC7344215

[B31] Wilson H. R. Cowan J. D. (1972). Excitatory and inhibitory interactions in localized populations of model neurons. Biophys. J. 12, 1–24. 10.1016/S0006-3495(72)86068-5 4332108 PMC1484078

[B32] Yeh F. (2017). Diffusion mri reconstruction in dsi studio. Adv. Biomed. MRI Lab. Natl. Taiwan Univ. Hosp. Available: http://dsi-studio. labsolver. org/Manual/Reconstruction# TOC-Q-Space-Diffeomorphic-Reconstruction-QSDR.

[B33] Yeo B. T. Krienen F. M. Sepulcre J. Sabuncu M. R. Lashkari D. Hollinshead M. (2011). The organization of the human cerebral cortex estimated by intrinsic functional connectivity. J. Neurophysiol. 106, 1125–1165. 10.1152/jn.00338.2011 21653723 PMC3174820

